# Characterization of ten highly polymorphic microsatellite loci for the intertidal mussel *Perna perna*, and cross species amplification within the genus

**DOI:** 10.1186/1756-0500-5-558

**Published:** 2012-10-08

**Authors:** Nelson C Coelho, Gerardo I Zardi, Gareth A Pearson, Ester A Serrão, Katy R Nicastro

**Affiliations:** 1 CCMAR, CIMAR-Laboratório Associado, University of Algarve, Campus de Gambelas, Faro, 8005-139, Portugal

**Keywords:** *Perna perna*, Brown mussel, Genetic diversity, Invasive

## Abstract

**Background:**

The brown mussel *Perna perna* (Linnaeus, 1758) is a dominant constituent of intertidal communities and a strong invader with multiple non-native populations distributed around the world. In a previous study, two polymorphic microsatellite loci were developed and used to determine population-level genetic diversity in invasive and native *P. perna* populations. However, higher number of microsatellite markers are required for reliable population genetic studies.

In this context, in order to understand *P. perna* origins and history of invasion and to compare population genetic structure in native versus invaded areas, we developed 10 polymorphic microsatellite markers.

**Findings:**

Described microsatellite markers were developed from an enriched genomic library. Analyses and characterization of loci using 20 individuals from a population in Western Sahara revealed on average 11 alleles per locus (range: 5–27) and mean gene diversity of 0.75 (range: 0.31 - 0.95). One primer pair revealed possible linkage disequilibrium while heterozygote deficiency was significant at four loci. Six of these markers cross-amplified in *P. canaliculus* (origin: New Zealand).

**Conclusions:**

Developed markers will be useful in addressing a variety of questions concerning *P. perna*, including dispersal scales, genetic variation and population structure, in both native and invaded areas.

## Findings

The genus *Perna* belongs to the Mytilidae (Mollusca; Bivalvia; Lamellibranchia; Mytiloida; Mytilidae), the family of “true mussels” which includes green and brown shell mussels from tropical, subtropical, warm and cold temperate regions [1]. Species within this genus are economically and ecologically important because they constitute an important source of human food [2,3]. They are dominant species on rocky shores often forming continuous beds in the intertidal and the shallow subtidal, providing microhabitats for many species [4].

The brown mussel *Perna perna* is a subtropical/tropical species widely distributed along the west coast of Madagascar, east African coast (from central Mozambique to False Bay), extending through the Gulf of Aden into the Red Sea, west coast of Africa (apart from the upwelling-influenced Benguela region on the west coast of South Africa) [5,6]; and from the Strait of Gibraltar to the Gulf of Tunis [7]. It is also present in Sri Lanka, southern India and in the Atlantic coast of South America where it was reported in Venezuela, Uruguay, and Brazil, as well as in the West Indies [7-9]. In Brazil, *P. perna* has been reclassified as an old introduction, most likely dating from the sixteenth century [10]. Moreover, it is reported as invasive in Western Australia [11] and in Texas, the Gulf of Mexico and southern Vera Cruz, where it was introduced via ballast water [12]. It has recently been reported for the first time on the southern Portuguese coast, suggesting a recent range expansion from Northern African shores [13]*.*

Despite the economic and ecological impacts of *P. perna*, only two microsatellite markers have previously been published for this species [14]. These markers were used to score individuals from 12 populations spanning the natural and introduced ranges of the brown mussel. To improve the accuracy of genetic studies we developed and characterized additional polymorphic microsatellite markers. The microsatellite markers developed in this study will also be valuable for food forensic uses and species genetic traceability through various steps in the food chain from producer to retailers. This will enable correct identification of food varieties, which is important in order to ensure quality, safety, authenticity and health for consumers. We describe ten highly polymorphic microsatellite loci for the mussel *P. perna*.

Total genomic DNA was extracted from 5 individuals collected in South Africa (Port Elizabeth, 33°58’47.81”S, 25°39’30.72”E) using a phenol-chloroform extraction method [15]. An enriched library was made by ecogenics GmbH (Zurich, Switzerland) from size selected genomic DNA ligated into SNX forward/SNX reverse-linker [16] and enriched by magnetic bead selection with biotin-labeled (CT)_13_, (GT)_13_, (TAC)_10_ and (GTAT)_7_ oligonucleotide repeats [17,18]. Of 528 recombinant colonies screened, 249 gave a positive signal after hybridization. Plasmids from 96 positive clones were sequenced and primers were designed for 32 microsatellite inserts, of which 26 were tested for polymorphism. Thirteen pairs of primers were excluded because they did not amplify in at least 16 of 20 individuals initially used for polymorphism tests and three were excluded because they resulted in an allelic pattern that was difficult to interpret. The resulting ten pairs of primers were selected as polymorphic loci and were further characterized using 20 *P. perna* individuals from one population from Western Sahara (Boujdour, 26°07’25”N, 14°29’57”W).

Polymerase chain reaction (PCR) was performed in volumes of 10 μl containing ±10 ng of DNA, 0.5 μM of each primer labelled with a florescent marker, 0.2 mM dNTPs (Bioline), 1.5 mM MgCl_2_, 3.0 μl of 5x PCR Buffer and 0.75 U of GoTaq Polymerase (Promega, Madison, WI). Cycling conditions consisted of an initial denaturing step of 5 min at 95°C, followed by 35 cycles of 30 s at 95°C, 30 s at annealing temperature (see Table 1 for locus optimization), 40 s at 72°C, and a final elongation step at 72°C for 20 minutes. All PCR reactions were performed in a GeneAmp 9700 thermocycler (PE Applied Biosystems). A DNA analyser (ABI PRISM 3130xl; Applied Biosystems) was used to analyse fragment lengths with the GeneScan Liz 500 size standard (Applied Biosystems). Raw allele sizes were scored with STRAND ( http://www.vgl.ucdavis.edu/informatics/STRand), binned using the R package MsatAllele [19], and manually reviewed for ambiguities. Observed (H_O_) and expected (H_E_) heterozygosities were estimated, and deviations from linkage and Hardy-Weinberg equilibria were tested using GENETIX software [20]. The number of alleles per locus ranged from 5 to 27 (Table 1). Expected and observed heterozygosities ranged from 0.31 to 0.95 and from 0.23 to 1.0, respectively, and significant heterozygote deficiency was detected in four loci after applying the q-value correction procedure (Table 1). High frequency of null alleles is likely at 3 of these loci (Perper01, Perper11 and Perper16), which was confirmed by further analysis using MICROCHECKER software [21].

**Table 1:** Characterization of ten microsatellite loci for brown mussel Perna perna

**Locus name (Genebank no.)**	**Primer sequences**	**Repeat motif**	**Clone size (bp)**	**PCR annealing (°C)**	***A***	**Size range (bp)**	**H**_**E**_	**H**_**O**_	**F**_**IS**_	**Cross amplification (positive/total)**
Perper-01	F-TGGAACTTAGGGCCTTCCTC	(AC)_13_	176	56	9	158-183	0.8075	0.45	0.46311**	3/5
(JX183697)	R-TCCAATTCTGTGAAATCATTGAC									
Perper-02	F-CCCGGTTTTAAGTGGTAGATG	(GT)_15_	113	57	6	110-137	0.3112	0.3000	0.06173	0/5
(JX183698)	R-AGCAAACGAAGGACAAATCG									
Perper-05	F-TCAGTGCCGCGTGATAATAC	(TG)_15_	127	59	16	111-150	0.885	1.000	−0.10465	4/5
(JX183699)	R-TCCAATTACGTTTGTTTTTGC									
Perper-08	F-AATGTTCAATGTCGACAGACTATG	(ACAG)_7_	129	56	5	110-126	0.7604	0.5789	0.26394*	0/5
(JX183700)	R-TTCTGAAGCACTGGATGTGG									
Perper-11	F-TTAACGTTGAAATCCGTGAGG	(GT)_9_…(GT)_13_	133	58	16	70-167	0.9044	0.4667	0.51000**	4/5
(JX183701)	R-CATTCCAATCCCACGCATAC									
Perper-16	F-TGTGATTTAAAGTTGGACTTGTTTC	(AC)_26_	134	56	8	104-130	0.5606	0.2353	0.60000**	0/5
(JX183702)	R-TGATTGGATCAAAATTAAACGTG									
Perper-20	F-ATGTCAATGTGCACAACACG	(ACAG)_15_	245	55	27	202-382	0.9525	0.9000	0.08065	0/5
(JX183703)	R-CGTGTATTGGCGACTTTTTATC									
Perper-26	F-CACCACCCTTACAAAGACGTG	(AC)_11_	102	53	11	92-113	0.845	0.7500	0.13767	5/5
(JX183704)	R-TTTCACTTGGCGATTAGTATGC									
Perper-27	F-CCCAATTTAACGGGAACAAC	(AC)_27_	167	56	13	103-193	0.8449	0.9474	−0.09459	4/5
(JX183705)	R-TTATCATCACCACTTTAAGTTACCC									
Perper-29	F-TTCCATTTCTAGACATCTCTGTCG	(AC)_11_	80	56	5	64-78	0.6763	0.95	−0.38314	5/5
(JX183706)	R-TCAGGTGACAGCAGCCTGAC									

Locus name and GeneBank accession number, primer sequence, motif repetition, clone size, PCR annealing temperature, number of alleles per locus, fragment size range, gene diversity, observed heterozygosity and inbreeding coefficient (* indicates significant values at p < 0.05 and ** at p < 0.001, after multiple tests correction). Cross amplification on *P. canaliculus* with five individuals.

We tested for linkage disequilibrium between all pairs of loci according to the procedure of Black and Krafsur [22]. The significance of the results was tested by permutation using 1000 replicates, and one pair (P20-P29) was significant at the 5% level (p = 0.045). Although all tested individuals were heterozygous for locus Perper05 (H_O_ = 1; Table 1), this result is identical to that expected under Hardy-Weinberg equilibrium (H_E_; Table 1) for this locus, as shown by its F_IS_ not significantly differing from zero. All markers were then tested for cross-amplification in five individuals of *P. canaliculus* from New Zealand, of which several amplifications were positive (Table 1).

Microsatellite markers have been developed for other species of the genus *Perna* (e.g. *P. canaliculus*[23]; *P. viridis*[24]) showing levels of polymorphism similar to those of the markers described in our study. The average allele number microsatellites are 8.8 and 11.7/locus for *P. canaliculus* and *P. viridis* respectively. Expected heterozygosity average at 0.78 and 0.69 for *P. canaliculus* and *P. viridis* respectively.

Deficiency in the number of heterozygotes observed (relative to Hardy-Weinberg expectation), with both allozyme and microsatellite studies have been documented in many marine molluscs e. g. [25-28], including species displaying separate sexes, e.g. *P. perna*. In this case, we cannot exclude species-specific, locus specific or population specific explanations for the results. Further genetic population structure analyses could shed light to this phenomenon in *P. perna*. Moreover, these ten microsatellite loci provide a useful tool to understand processes influencing species boundaries, such as range expansions outside the native distribution of *P. perna* populations, and compare diversity and differentiation scales in invasive and native populations.

### Availability of supporting data

The microsatellite sequences are available through the National Center for Biotechnology Information (see http://www.ncbi.nlm.nih.gov/). The accession numbers on the repository are the following (see also Table 1):

Perper-01- JX183697; Perper-02 -JX183698; Perper-05 -JX183699; Perper-08 -JX183700; Perper-11 -JX183701; Perper-16 -JX183702; Perper-20 -JX183703; Perper-26 -JX183704; Perper-27 -JX183705 and Perper-29 -JX183706.

## Competing interests

The authors declare they have no competing interests.

## Authors’ contributions

All authors participated in the design and implementation of the study, supervision of the work and processing interpretation of the results. CNC, NKR and ZGI participated in data analysis, microsatellite marker validation and drafted the manuscript. All authors read and approved the final manuscript.
